# The evidence of indirect transmission of SARS-CoV-2 reported in Guangzhou, China

**DOI:** 10.1186/s12889-020-09296-y

**Published:** 2020-08-05

**Authors:** Chaojun Xie, Hongjun Zhao, Kuibiao Li, Zhoubin Zhang, Xiaoxiao Lu, Huide Peng, Dahu Wang, Jin Chen, Xiao Zhang, Di Wu, Yuzhou Gu, Jun Yuan, Lin Zhang, Jiachun Lu

**Affiliations:** 1 The Institute for Public Health, Guangzhou Medical University, 195 Dongfengxi Road, Guangzhou, 510182 PR China; 2 Guangzhou Center for Disease Control and Prevention, 1Qide Road, Guangzhou, 510440 PR China; 3 State Key Lab of Respiratory Disease, The First Affiliated Hospital, Guangzhou Medical University, 195 Dongfengxi Road, Guangzhou, 510182 PR China; 4 Department of English and American Studies, Faculty of Languages and Literatures, Ludwig Maximilian University (LMU), Munich, Germany; 5 Baiyun District Center for Disease Control and Prevention, Guangzhou, PR China

**Keywords:** Novel coronavirus disease, Severe acute respiratory syndrome coronavirus 2, Snot-oral transmission, Indirect transmission

## Abstract

**Background:**

More than 2 months have passed since the novel coronavirus disease 2019 (COVID-19) first emerged in Wuhan, China. With the migration of people, the epidemic has rapidly spread within China and throughout the world. Due to the severity of the epidemic, undiscovered transmission of COVID-19 deserves further investigation. The aim of our study hypothesized possible modes of SARS-CoV-2 transmission and how the virus may have spread between two family clusters within a residential building in Guangzhou, China.

**Methods:**

In a cross-sectional study, we monitored and traced confirmed patients and their close contacts from January 11 to February 5, 2020 in Guangzhou, China, including 2 family cluster cases and 61 residents within one residential building. The environmental samples of the building and the throat swabs from the patients and from their related individuals were collected for SARS-CoV-2 and tested with real-time reverse transcriptase polymerase chain reaction (RT-PCR). The relevant information was collected and reported using big data tools.

**Results:**

There were two notable family cluster cases in Guangzhou, which included 3 confirmed patients (family No.1: patient A, B, C) and 2 confirmed patients (family No.2: patient D, E), respectively. None of patients had contact with other confirmed patients before the onset of symptoms, and only patient A and patient B made a short stop in Wuhan by train. Home environment inspection results showed that the door handle of family No.1 was positive of SARS-CoV-2. The close contacts of the 5 patients all tested negative of SARS-CoV-2 and in good health, and therefore were released after the official medical observation period of 14-days. Finally, according to the traceability investigation through applying big data analysis, we found an epidemiological association between family No.1 and family No.2, in which patient D (family No.2) was infected through touching an elevator button contaminated by snot with virus from patient A (family No.1) on the same day.

**Conclusions:**

Contaminants with virus from confirmed patients can pollute the environment of public places, and the virus can survive on the surface of objects for a short period of time. Therefore, in addition to the conventional droplet transmission, there is also indirect contact transmission such as snot-oral transmission that plays a crucial role in community spread of the virus.

## Background

In late December 2019, an outbreak of the novel coronavirus disease 2019 (COVID-19) caused by the severe acute respiratory syndrome coronavirus 2 (SARS-CoV-2) was reported by the local health facilities in Wuhan, China [1, 2]. The epidemic has been spreading to many other Chinese cities [3–5]. As the spread escalated, the World Health Organization (WHO) declared that the SARS-CoV-2 outbreak constitutes a “Public Health Emergency of International Concern” on January 30, 2020, and the epidemic has escalated to a pandemic since March 11, 2020.

With the information from epidemiological investigation and clinical manifestation accumulating, evidence indicated that there existed person-to-person transmission of COVID-19 [6, 7]. In order to elucidate the reason for the rapid spread of the disease, the researchers examined whether the virus could survive in external environment and found detectable nucleic acid of SARS-CoV-2 in environmental sample gathered from South China Seafood Market in Wuhan, the place where the virus first broke out. Other studies also showed that the virus was also found in patients’ feces and urine [8]. The pre-existing evidence suggests that contaminants from patients can pollute the environment of public places and indirectly indicates the possibility of contact transmission. Due to the fact that the Chinese government had invested a lot of resources and adopted various measures to manage the impact of the outbreak, the number of confirmed COVID-19 cases and suspected cases has begun to decline slowly since 15 February 2020, and there has been no new cases for days in several provinces and cities [9]. But the epidemic has not completely come to an end and we still need to remain vigilant continuously especially because of the migration of large populations and the gradual reopening of public places. New reports have shown that the virus continues to spread, yet we still have very few understandings of the characteristics of the virus, and therefore need further investigation. As reported in this study, there were two interesting family cluster cases reported in Guangzhou, providing the evidence of snot-oral indirect transmission of SARS-CoV-2.

## Methods

A cross-sectional study was conducted to investigate the possible modes of SARS-CoV-2 transmission between 2 family clusters within the same residential building in Guangzhou, China. Between January 11, 2020 and February 5, 2020, two family cluster cases included 5 confirmed patients reported in one residential building. We investigated cases, close contacts, the residents,security guards and janitors of this building. On January 30, we sampled aerosol and surface samples from the elevator and the houses of the two families. On February 1, we sampled blood samples and throat swabs from the residents of this building, and throat swabs collected from the security guards and janitors. On February 3, 2020, the second batch of surface samples from the elevator and the houses of the two families were collected. The environmental samples of the building and the throat swabs from the patients and their related individuals were tested with real-time reverse transcriptase polymerase chain reaction (RT-PCR) for SARS-CoV-2. The relevant information was collected and analyzed using big data tools.

### Data collection

All information of traceability investigation was collected using big data tools and reported by Guangzhou Center for Disease Control and Prevention (Guangzhou CDC), and the field epidemiological investigations were conducted by the staff of Guangzhou municipal and Baiyun district centers for disease control and prevention based on the “questionnaire on individual case of COVID-19 cases” and the “questionnaire on Individual case of suspected COVID-19 cases”. The pharyngeal swabs, blood samples, aerosol samples and surface samples were collected by medical staff in hospitals or during the field investigation, and safely sent to Guangzhou Centre for Disease Control and Prevention. The real-time reverse-transcriptase–polymerase-chain-reaction (RT-PCR) assay was performed to confirm the infection caused by the SARS-Cov-2 virus. The informed consent was obtained from each participant and this epidemiological study was approved by the Ethical Committee of Guangzhou Centre for Disease Control and Prevention.

### Study definitions

#### Big data tools

The “big data tools” is a database containing information about monitored and traced cases and close contacts accumulated and managed by Guangzhou CDC, from which we get the information of 2 family cluster cases and 61 residents of same residential building from January 11 to February 5, 2020 in Guangzhou, China.

#### Sample collection

3 aerosol samples and 21 surface samples were collected from the elevator and the houses of the two families. In addition to collecting throat swabs from 5 confirmed patients, there were 61 blood samples and 61 throat swabs collected from the residents of this building, and 14 throat swabs collected from security guards and janitors, as shown in Table 2.

#### Viral nucleic acid test

Laboratory confirmation of the SARS-CoV-2 by RT-PCR assay (Shanghai BioGerm Medical Biotechnology) was conducted in Guangzhou Center for Disease Prevention and Control (Guangzhou CDC). The RT-PCR assay was performed in accordance with the protocol established by the World Health Organization. SARS-CoV-2 nucleic acid testing was performed using RT-PCR assay according to the National Health Commission guidelines for laboratory testing of pneumonia with novel coronavirus infection [10, 11].

#### The COVID-19 patient

The case is determined by a positive result by real-time reverse-transcriptase–polymerase-chain-reaction (RT-PCR) assay of the SARS-Cov-2 virus in patient’s pharyngeal and anal swab specimens. Only the laboratory-confirmed patients were included in the final analysis.

#### Asymptomatic patient

An asymptomatic case is defined as someone who shows no clinical symptoms within 14 days before the diagnosis, tests positive for SARS-CoV-2 on RT-PCR or serum-specific IgM antibodies, and is identified through close contact screening, cluster epidemic investigation and traceback investigation.

#### The exposure

The exposure is defined as the following situations within 14 days before the onset of illness applicable to individuals: 1) traveling to or living in Wuhan or other regions with severe epidemics abroad; 2) having contact with SARS-CoV-2 infected individuals whose nucleic acid test was positive or with patients who had fever or respiratory symptoms coming from Wuhan or other regions abroad that have been severely affected by the epidemic; 3) having been to 2 or more cases occurred fever or respiratory symptoms in a small area (such as home, office, school class, workshop, construction site, etc.).

#### Cluster outbreak

The cluster outbreak is defined as 2 or more presumptive confirmed cases reported with fever or respiratory symptoms within 14 days after having been in the same confined space (such as home, office, school class, workshop, construction site, etc.), which provides the possibility of interpersonal transmission and being infected due to co-exposure.

#### Close contact

Close contact refers to an individual who has not taken effective protection when in proximity of suspected or confirmed cases 2 days before the onset of symptoms or 2 days before the collection of asymptomatic specimens.

### Statistical analysis

We setup the EXCEL databases to include all questionnaires, clinic and laboratory data of two family COVID-19 patients. The continuous variables were shown as medians and interquartile ranges (Q1-Q4), Median (IQR), or medians and ranges, Median (min-max). The categorical variables were summarized as counts and percentages, no. (%). All the figures were drawn using Graphad Prism 8 software, and all the analyses were performed using SPSS software (Statistical Package for the Social Sciences, version 26.0).

## Results

### Characteristics of two family cluster cases

In the cross-sectional study of COVID-19 in Guangzhou, we found that there were two notable family cluster cases, which included 3 (family No.1: patient A, B and C) and 2 (family No.2: patient D and E) cases of COVID-19 patients, respectively. As listed in Table 1, patient A was male and 70 years old; patient B and C were female and were 68 and 38 years old. Patient D was a 64 year old female, and patient E was male and 66 years of age. All patients had fever at onset, except for patient E, who was symptomless.

**Table 1:** The basic information of 5 patients with COVID-19

Patients	Occupation	Symptoms	Temperature	Blood leukocyte count (*10^9^/L)	Lymphocyte count(*10^9^/L)	Lymphocyte percentage(%)	Neutrophil percentage(%)	CT	Onset date	Admission date	Out date
Patient A	Retiree	Fever, running nose	38 °C	4.25	0.74	17.4	75.1	B +	Jan13 2020	Jan24 2020	Feb13 2020
Patient B	Retiree	Fever, dry cough	37.5 °C	6.05	2.1	34.7	56.8	B -	Jan23 2020	Jan24 2020	Feb23 2020
Patient C	Civil servants	Fever	38.5 °C	5.66	1.47	26.04	65.4	B +	Jan19 2020	Jan24 2020	Feb8 2020
Patient D	Retiree	Fever	37.8 °C	5.42	1.21	22.34	66.44	B +	Jan21 2020	Jan27 2020	Feb21 2020
Patient E	Retiree	–	Normal	Normal	Normal	Normal	Normal	R up +	asymptomatic	Jan27 2020	Feb21 2020

Note: Age (range, 38–70 years); *CT* Computerized tomography, *B* Bilateral lung, *R up* the upper lobe of the right lung

### Information of family No.1

**Fig. 1:**
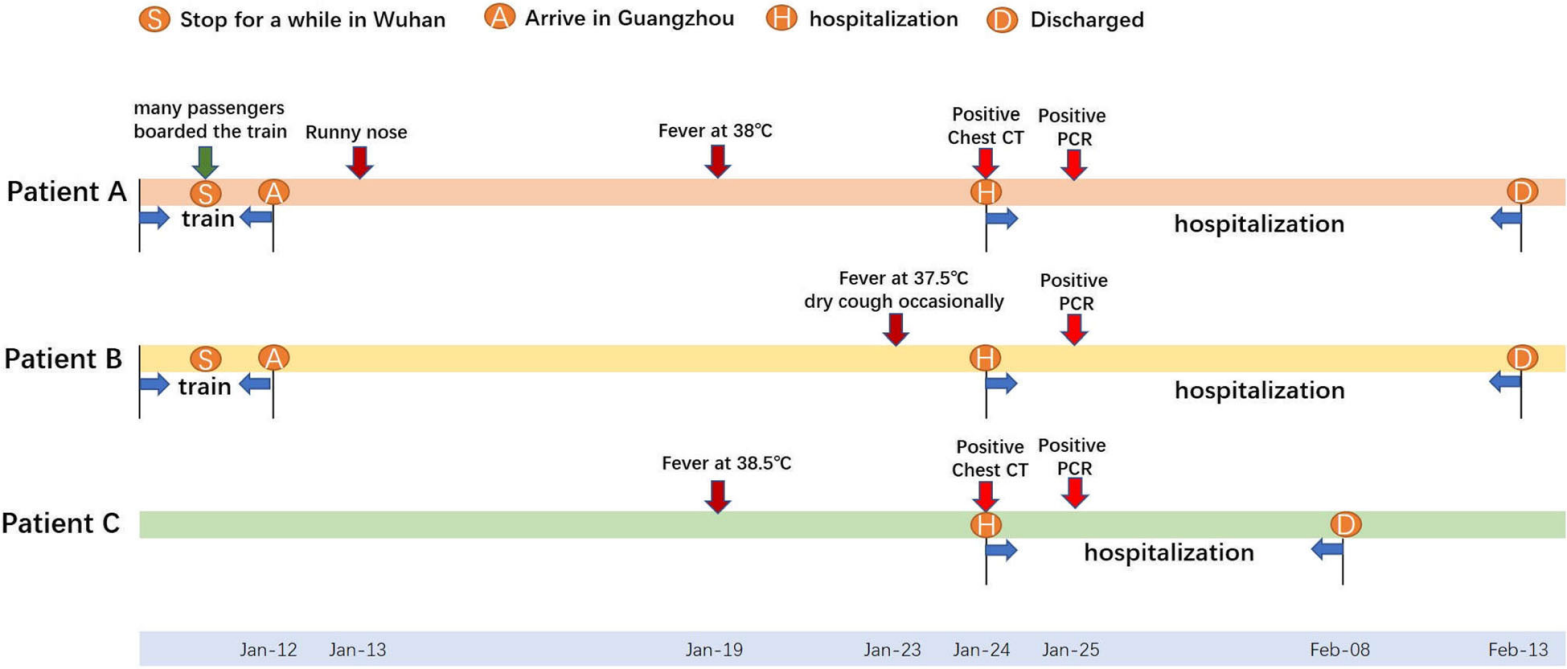
The diagram of epidemiological survey in family No.1

Patient A, 70 years old, developed a fever at 38 °C accompanied with runny nose on January 19th, 2020, as shown in Fig. 1. Patient B, patient A’s wife, developed a fever at 37.5 °C with an occasional dry cough on January 23th. Patient C, their daughter, developed a fever at 38.5 °C without other symptoms on January 19th. On January 24th, the three patients visited Jinshazhou Hospital of Guangzhou Chinese Medicine University for treatment. The chest CT of patient A showed bilateral lung inflammation and emphysema and blood test showed decreased lymphocyte count. The chest CT result of patient C also showed bilateral lung inflammation, while that of patient B was normal. On the night of the same day, they were admitted to the isolation ward of hospital as suspected cases of COVID-19. At 9:00 a.m. on January 25, their throat swabs were collected by Guangzhou CDC and tested for SARS-CoV-2 virus nucleic acid test, all of which were reported to be positive on the same day, so they were immediately transported by ambulance to the designated COVID-19 hospital for isolation treatment. These three patients were in mild severity. After isolation and treatment in the designated hospital, they were cured and discharged. A total of 18 individuals were identified as close contacts of this family by Guangzhou CDC and so far, all of them were healthy without any symptoms and thus are all relieved of medical observation.

### Information of family No.2

**Fig. 2:**
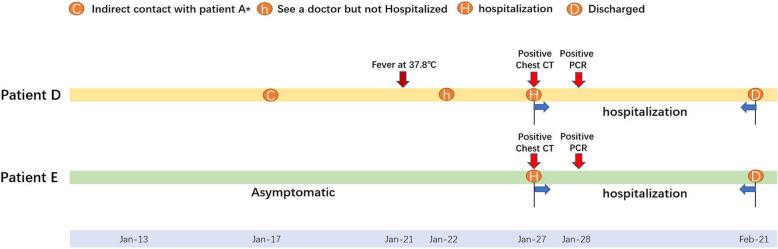
The diagram of epidemiological survey in family No.2

As described in Fig. 2, Patient D, a 64-year-old woman, experienced the onset of fever at 37.8 °C without other symptoms on January 21th, and went to Jinsha Street Community Health Service Center for treatment on January 22th. Patient E, patient D’s husband, without any respiratory symptoms, he went to Jinshazhou Hospital of Guangzhou Chinese Medicine University with his wife on January 27th. The chest CT test of patient D showed bilateral lung inflammation and that of patient E showed inflammation in the upper lobe of the right lung. Both patients were admitted to the isolation ward of hospital as suspected cases of COVID-19 on the same day. On January 28th, the outcomes of virus nucleic acid test were both positive of SARS-CoV-2 so that they were transported by ambulance to the designated hospital of COVID-19 for isolation treatment on January 29th. They were both mild patients in stable condition. After isolation and treatment in the designated hospital, they were cured and discharged. Three other individuals were identified as close contacts of this family by Guangzhou CDC and so far, all were in good health without any symptoms so that they were all relieved of medical observation.

### The results of viral nucleic acid test

**Fig. 3:**
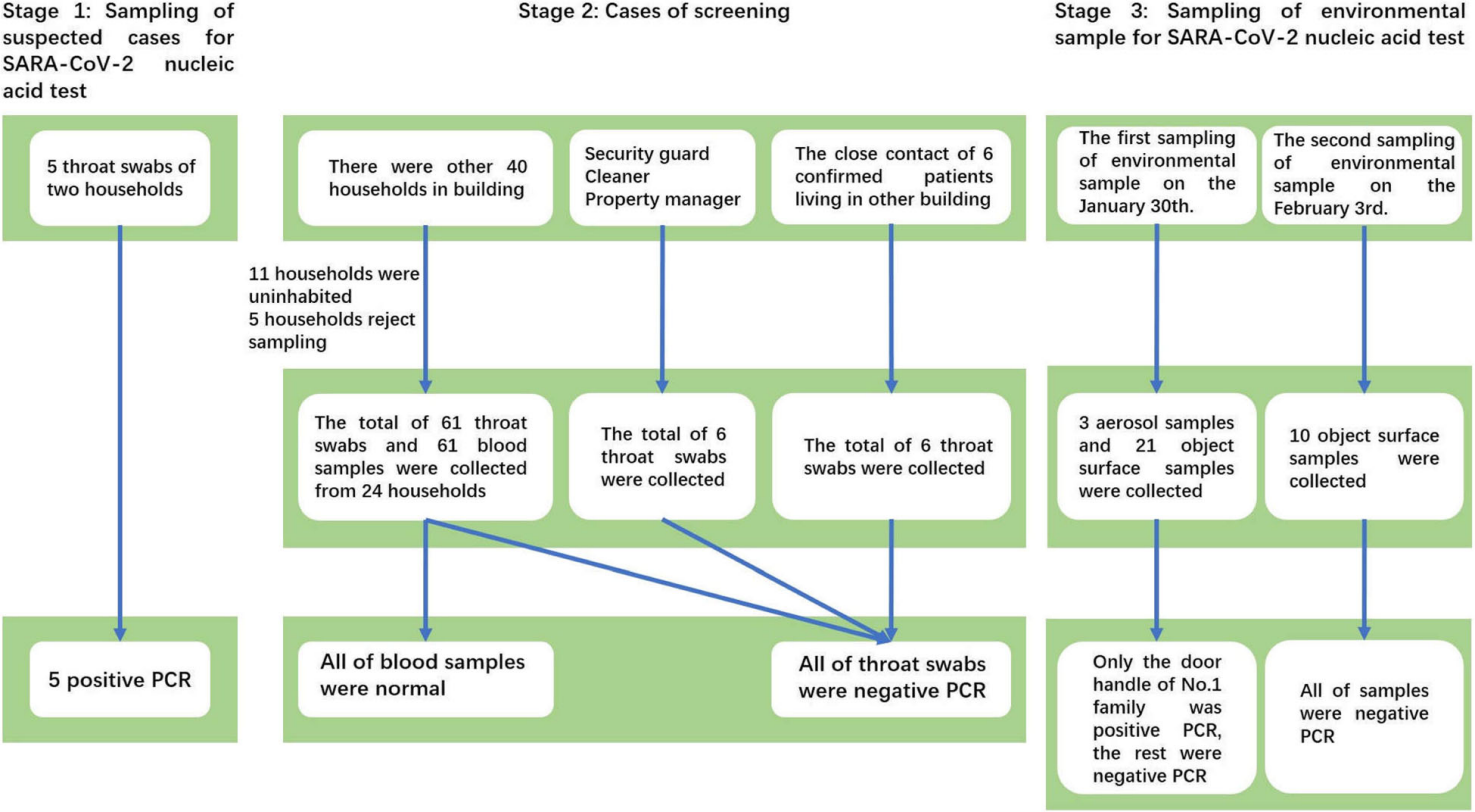
The graphical of detecting steps of SARS-CoV-2 among the patients and their close contacts, and their surroundings

**Fig. 4:**
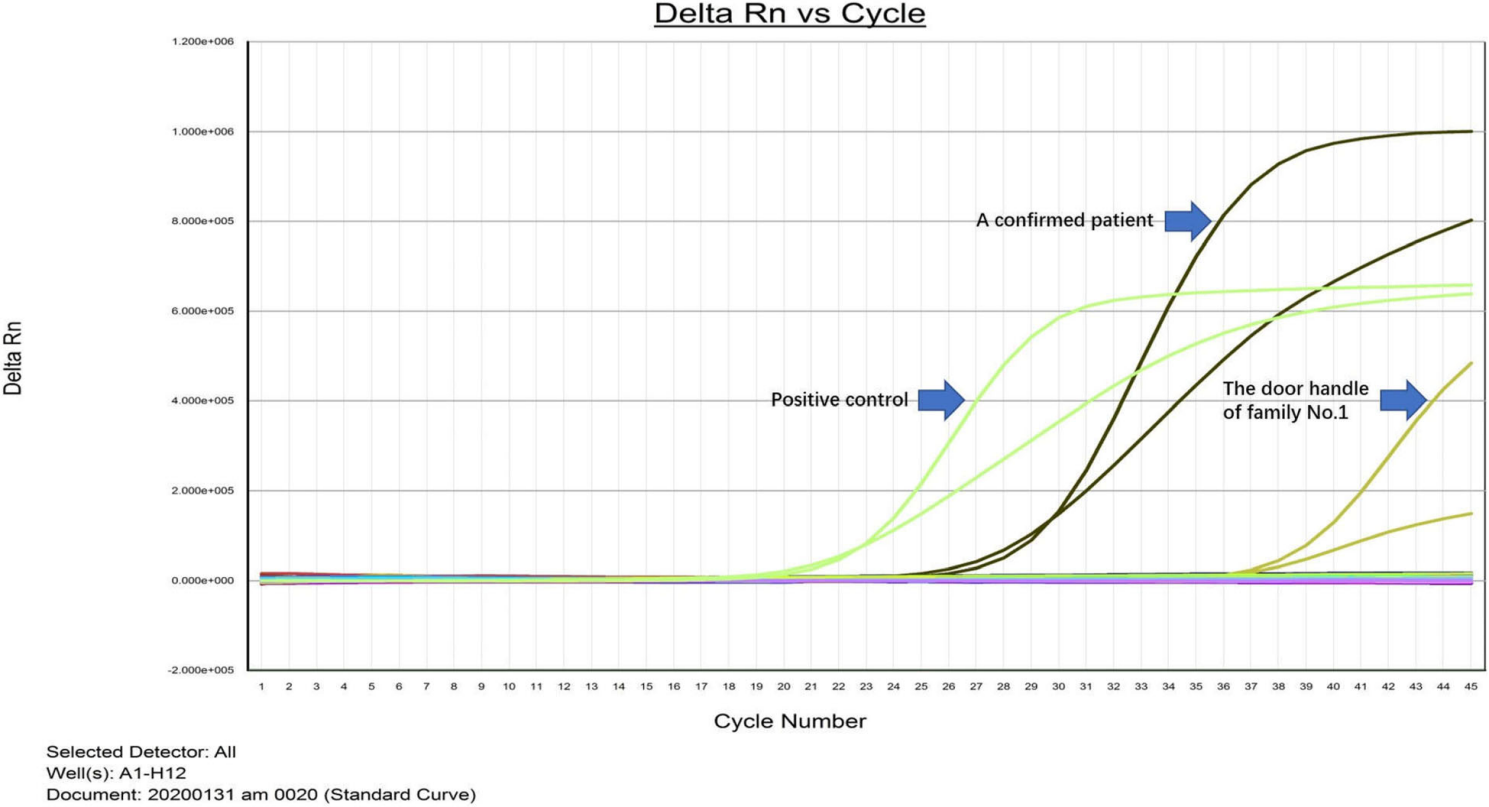
The result of SARS-CoV-2 viral nucleic acid test for the door handle

**Fig. 5:**
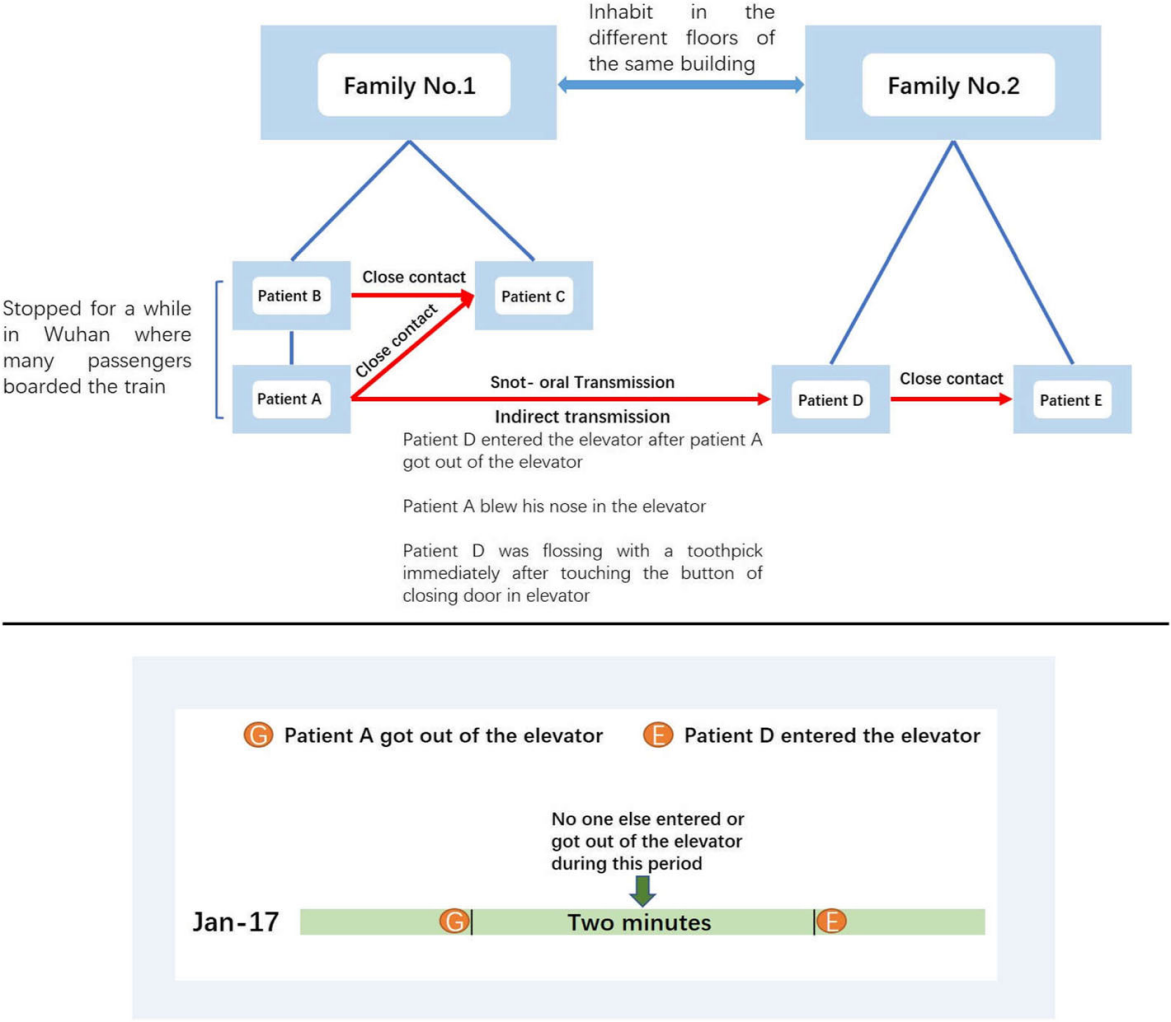
The epidemiological investigation and their relationship between family No.1 and family No.2

As presented in Figs. 3, 5 patients from the above two families were positive of SARS-CoV-2, while the test results of throat swabs and blood samples collected from relevant individuals were all negative, as listed in Table S 1 and S 2. Of all environmental samples, only the door handle of family No.1 tested positive of SARS-CoV-2(shown in Fig. 4), and the rest were negative that listed in Table 2.

**Table 2:** The outcome of environmental samples tested

Sample number	Sampling area	Sample type	Outcomes
The first sampling of environmental samples on the 30 January, 2020.			
1	Up and down Button of 01 floor outside of elevator	Object surface swab	Negative
2	Button of close and open inside of elevator	Object surface swab	Negative
3	Button of 01-B1 floor inside of elevator	Object surface swab	Negative
4	Button of 02–11 floor inside of elevator	Object surface swab	Negative
5	The ground of elevator	Object surface swab	Negative
6	The wall of elevator	Object surface swab	Negative
7	Both sides of elevator door	Object surface swab	Negative
8	The door of elevator	Object surface swab	Negative
9	Button of 10 floor	Object surface swab	Negative
10	The door handle of No.1 family	Object surface swab	Positive*
11	Button of 11 floor	Object surface swab	Negative
12	The door handle of No.2 family	Object surface swab	Negative
13	The door handle of the big door of building	Object surface swab	Negative
14	Button of 02 floor outside of elevator	Object surface swab	Negative
15	Button of 03 floor outside of elevator	Object surface swab	Negative
16	Button of 04 floor outside of elevator	Object surface swab	Negative
17	Button of 05 floor outside of elevator	Object surface swab	Negative
18	Button of 06 floor outside of elevator	Object surface swab	Negative
19	Button of 07 floor outside of elevator	Object surface swab	Negative
20	Button of 08 floor outside of elevator	Object surface swab	Negative
21	Button of 09 floor outside of elevator	Object surface swab	Negative
22	Inside the elevator	aerosol	Negative
23	The house of No.1 family	aerosol	Negative
24	The house of No.2 family	aerosol	Negative
The second sampling of environmental samples on the 3 February, 2020.			
1	Button inside of elevator	Object surface swab	Negative
2	Button of 08 floor outside of elevator	Object surface swab	Negative
3	First floor stair railing	Object surface swab	Negative
4	The door handle of No.2 family	Object surface swab	Negative
5	Third floor stair railing	Object surface swab	Negative
6	First floor stair railing outside of big door of building	Object surface swab	Negative
7	The door handle of 702 household	Object surface swab	Negative
8	The door handle of 1102 household	Object surface swab	Negative
9	The door handle of No.1 family	Object surface swab	Negative
10	Button of 02 floor outside of elevator	Object surface swab	Negative

### Epidemiological investigation

All three members of family No.1 said that they had no contact with COVID-19 patients nor individuals from Hubei province. However, patient A and patient B had traveled to Guangzhou by train from other province to visit their children (patient C). The train stopped in Wuhan for several minutes. That stop was a crowded and compact hub where many passengers boarded the train. Therefore, as shown in Fig. 5, it was inferred that patient A and patient B were infected through close contact to passengers who might be unknown patients of COVID-19. Later they passed on the COVID-19 to their daughter (patient C) as close family contact. Both members of family No.2 said that they had no contact with the patients in family No.1 or individuals from Hubei province, and had no link to Wuhan city.

### Epidemiological relationship between family No.1 and family No.2

According to the traceability investigation through applying the big data tools, we found that patient A had a bad habit in personal hygiene that he often blew nose using his own hand, which was what he again did before touching the button of closing door in elevator. As shown in Fig. 5, on January 17th, patient A blew nose using his own hand before touching the button of closing door in elevator, then 2 min after patient A got out of the elevator, patient D entered the same elevator and touched the same button. The most important thing is that patient D immediately flossed with a toothpick after touching the elevator button. Therefore, it was speculated that patient D (family No.2) was infected for COVID-19 by means of snot-oral indirect transmission of touching the button of elevator contaminated by snot with virus from patient A (family No.1).

## Discussion

Although the epidemic of COVID-19 has been going on for months, the epidemiological characteristics of the SARS-CoV-2 virus are not yet fully understood. With the import of COVID-19 cases, there were many new local patients in the cities outside of Hubei province [5, 12]. Some cities have a high proportion of clustering cases, such as Beijing, where, as of February 11, a total of 77 clustered cases involving 251 patients accounted for 71% of a total of 352 patients [13]. A point worth noting about this epidemic is that some local cases have no clear source of infection in the cities outside of Hubei province.

Recently, there were several reports that the new coronavirus could spread via droplets, contact and natural aerosols from human-to-human [1, 4, 14, 15], causing a high possibility of a pandemic. As more and more new cases with COVID-19 are reported worldwide [16, 17], it seems to be a gloomy reality. To contain the spread of the COVID-19 epidemic without delay, a deeper understanding of the SARS-CoV-2 virus should be presented [18]. To reduce the impact and spread of the disease, it is essential to limit human-to-human transmission to reduce secondary infections among close contacts and health care workers.

As reported in this study, we found two family clusters infected with SARS-CoV-2 in the same building in Guangzhou, China. Through testing the external environment samples, we found that the sample taken from door handle of family No.1 tested positive of SARS-CoV-2, which indicated that contaminants with virus from confirmed patients can pollute the environment of public places. Furthermore, the patient D of family No.2 was infected via the snot-oral indirect transmission, indicating that SARS-CoV-2 virus can survive in the environment for at least a short period of time. As previously reported by Zou LR et al., higher viral loads were detected soon after symptom onset, with higher viral loads detected in the nose than in the throat [19, 20], which further demonstrated that indirect contact transmission by means of snot-oral Transmission might be an effective way to spread the epidemic disease. For the surface survival of virus, SARS-CoV-2 can remain viable and infectious on surfaces up to days, so common surface transmission of the virus is highly possible [21]. Although the elevator buttons were detected as negative for viral nucleic acid, the possible reason is that the buttons have been used many times and the time interval from contamination to sampling is too long. During this period, the interior of the elevator has been disinfected several times. With the return to work and the gradual opening of public places, the migration of large population is a huge challenge for prevention and control of the epidemic. Therefore, it is also still important for the prevention and control of the epidemic to pay attention to personal hygiene, taking measures such as wearing a facemask, washing hands frequently and not touching nose and mouth before washing hands and so on, as well as disinfection of public places in the coming time period.

Our study had some obvious limitations. First, on January 17, no samples were collected on the day of the elevator button pollution and our elevator button sampling took place on January 30. Second, according to the weak positive test of patient A’s home handle and his poor hygiene habits, it is our inference that the infection of patient D was caused by the transmission of the elevator button polluted by patient A’s nose. Third, we cannot exclude the possibility of transmission of the virus by unknown infected persons, such as asymptomatic carriers [22].

## Conclusions

In summary, this study provides direct evidence substantiating that SARS-CoV-2 can infect other individuals by means of snot-oral transmission as one mode of indirect contact transmission. This finding is of significance for the prevention and control of COVID-19 and the formulation of the public health policies and measures. Based on previous reports and the evidence provided in this study, it is important to pay attention to personal hygiene and disinfection in public places.

## Supplementary information

**Additional file 1: Table S1.** The outcome of whole blood samples tested. **Table S2.** The outcome of throat swabs tested.

## Data Availability

The datasets used and/or analyzed during the current study are available from the corresponding author on reasonable request.

## Abbreviations

SARS-CoV-2: Severe acute respiratory syndrome coronavirus 2; COVID-19: Corona virus disease 2019
RT-PCR: Real-time reverse transcriptase polymerase chain reaction
IQR: Interquartile ranges
CT: Computerized tomography
B: Bilateral lung
R_up_: Upper lobe of the right lung
